# A Review of GPS Trajectories Classification Based on Transportation Mode

**DOI:** 10.3390/s18113741

**Published:** 2018-11-02

**Authors:** Xue Yang, Kathleen Stewart, Luliang Tang, Zhong Xie, Qingquan Li

**Affiliations:** 1 Faculty of Information Engineering, China University of Geosciences, Wuhan 430074, China; xiezhong68@gmail.com; 2 Department of Geographical Sciences, University of Maryland, College Park, MD 20742, USA; stewartk@umd.edu; 3 State Key Laboratory of Information Engineering in Surveying, Mapping, and Remote Sensing, Wuhan University, Wuhan 430079, China; tll@whu.edu.cn; 4 College of Civil Engineering, Shenzhen University, Shenzhen 518060, China; liqq@szu.edu.cn

**Keywords:** GPS data, trajectory generation, movement parameters, transportation mode

## Abstract

GPS trajectories generated by moving objects provide researchers with an excellent resource for revealing patterns of human activities. Relevant research based on GPS trajectories includes the fields of location-based services, transportation science, and urban studies among others. Research relating to how to obtain GPS data (e.g., GPS data acquisition, GPS data processing) is receiving significant attention because of the availability of GPS data collecting platforms. One such problem is the GPS data classification based on transportation mode. The challenge of classifying trajectories by transportation mode has approached detecting different modes of movement through the application of several strategies. From a GPS data acquisition point of view, this paper macroscopically classifies the transportation mode of GPS data into single-mode and mixed-mode. That means GPS trajectories collected based on one type of transportation mode are regarded as single-mode data; otherwise it is considered as mixed-mode data. The one big difference of classification strategy between single-mode and mixed-mode GPS data is whether we need to recognize the transition points or activity episodes first. Based on this, we systematically review existing classification methods for single-mode and mixed-mode GPS data and introduce the contributions of these methods as well as discuss their unresolved issues to provide directions for future studies in this field. Based on this review and the transportation application at hand, researchers can select the most appropriate method and endeavor to improve them.

## 1. Introduction

Technological advances in navigation and positioning, together with the development of location-based services, have enabled smart devices (i.e., smartphones, tablets, wearables) to become an indispensable part of daily human actions, activities and interactions [1,2,3]. GPS positional data generated from these smart devices provides the foundation for revealing a vast variety of location-based activities [4,5], including, for example, human daily activity pattern mining [6]; behavior analysis [7]; human health monitoring [8,9]; road/pedestrian network generation and updating [10,11,12]; transportation monitoring [13]; land-use detection [14]; space optimization [15,16]; route planning and point of interest recommendation [17,18], etc. Specially, for location-based services, for example, the system may send customized advertisements and real-time traffic information to travelers based on the knowledge of their transportation mode and location. For transportation science, travel behavior research is undertaken based on GPS data provided by travelers. To understand these travel behaviors, GPS data classification based on transportation mode is an important first step. In addition, for other knowledge mining research, GPS data with specific transportation mode data provides a rich set of information, such as information about vehicles trajectories; and from pedestrian trajectories [19,20]. Therefore, for these GPS data-driven applications and developments, data classification based on the underlying transportation mode plays an important role in providing task-focused data support.

**Figure 1:**
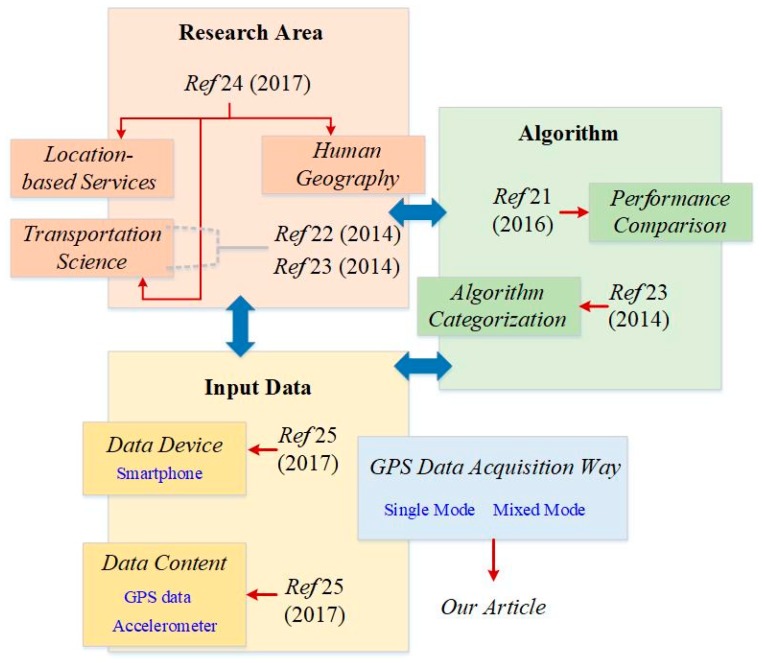
Overview of recent review papers for transportation mode detection.

The key to GPS trajectories classification based on the underlying transport mode is recognizing the transport mode of each GPS point, GPS segment, or an entire GPS trajectory. In recent decades, research on transportation mode detection from GPS data has been widely discussed, and a considerable number of approaches for detecting transport mode have been proposed. These methods, vary by input variables; and classification algorithm. Authors in Ref. [21] compared the relative performance of different classification algorithms for the detection of transportation modes by using the same GPS data set. In Ref. [22], researchers reviewed some of the methods for GPS data classification based on transportation mode from the perspective of GPS surveys including travel behavior change monitoring, route choice, traffic safety, etc. Similarly, the authors in Ref. [23] summarized the methodologies of GPS data classification based on transport mode into three main categories: machine learning approaches, probability-based methods that use fuzzy logic rules and probability matrices, and criteria-based method. Although these two review papers [22,23] offered a good overview of what methods were used in transportation science, the solutions provided by research fields are not limited to transportation science. As an extension of the review study, researchers in Ref. [24] given a detailed summary of the solutions for transportation mode detection based on GPS data in different research field including location-based services, transportation science, and human geography. They also discussed and compared the methods of transportation mode detection by using accelerometer-only data set, GPS-only data, and GPS fused with accelerometer data in different research areas. In addition, the methods of transportation mode detection by using smartphone are also reviewed by researchers from the aspects of the type and number of used input data, transportation mode categories, and the algorithm used for the classification task [25]. In particular, to help us gain an overview of what methods will be used as we face different aims in a specific field, methods of transportation mode detection based on above review papers [21,22,23,24,25] are categorized into three groups: research area, algorithm, and input-data, as shown in Figure 1. Each group has the representative articles marked with the publication date (see Figure 1).

**Figure 2:**
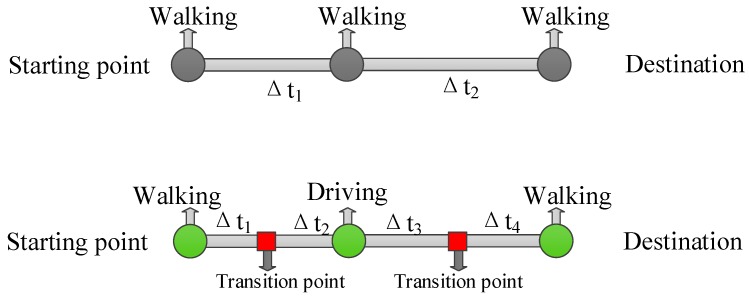
Analysis of the use of transportation mode of moving objects.

Unlike previous review works that summary and discuss the methods of GPS data classification from the aspects of research area, algorithms, and input-data, we provide a systematic overview of GPS data classification methods from the perspective of GPS data acquisition way. Approaches on GPS data classification based on underlying transport mode are classifies into two main groups, single-mode methods and mixed-mode methods, according to the way that GPS trajectories are generated. As part of our review, we discuss the unsolved issues with existing methods and provide future directions for classifying single-mode and mixed-mode GPS trajectories. In summary, the contributions of this paper include: (1) to provide an overview of classification methods of GPS data from a data processing standpoint and extend the review study of GPS data classification from the perspective of input-data (see Figure 1); (2) the challenges and unsolved issues with the existing approaches for each type of GPS trajectories are discussed and summarized in this article.

## 2. Preliminaries

### 2.1. Discussion: GPS Data Acquisition and Characteristics

GPS data as a major part of spatial big data [26] records the position, gathering time, heading, speed, and other movement attributes of moving objects. At present, GPS trajectories can be obtained from a number of sources: volunteers (e.g., OpenStreetMap, Geo-life) and public service platforms (e.g., taxi trajectories collected by taxi companies in Wuhan or other cities in China), etc. In Ref. [27], authors proposed that the GPS position data with timestamp can be generated through two ways: passive data collection, and active data collection. Passive data collection means that data is collected using inbuilt sensor technologies. Instead, active data collection indicates that data is generated by active and sporadic user input. Different data collection methods bring different influences on data quality, data completeness, data information, etc. Researchers compared the similarities and differences of transport data collected in two ways (e.g., passive and active way) from four aspects, which are data quality, safety issues, data completeness, and user requirements [28]. We summarize the differences of GPS data acquisition in two ways from position precision, real-time, and movement information (i.e., transportation mode), as shown in Table 1.

Based on Table 1, we can see that the transportation mode of GPS data collected either passively or actively can vary. For GPS data collected by the passive way, whether the transportation mode of GPS data is known depends on the application requirement. For example, the transportation mode of some vehicle GPS data is known if it is collected by taxi or bus monitoring system. But it is may be unknown if GPS data is generated from users’ smartphones when they are using the related location-based services. Compared with passive data collection, transportation mode of GPS data using active way mainly depends on users’ behavior. For example, some volunteers will give a detailed description of transportation mode when they upload their traveling route data (e.g., a part of GPS data in the OSM website and Geo-life dataset), while others just share their data without a description of transportation mode. For most GPS data that currently is used, data collection methods are not limited to either passive or active data collection but can use a mix of both techniques [29,30]. Thus, GPS trajectories classification based on transportation mode is very necessary for subsequent GPS data-based research.

### 2.2. A Macroscopic Classification of GPS Data Based on Trajectories Generation Way

The GPS trajectory of a moving object is a set of sampled positions with a time stamp and other related movement information (e.g., speed and moving direction). Essentially, GPS tracking data express the continuous motion of moving object in discrete form. In reality, moving objects may change their transport mode through time (e.g., people). As shown in Figure 2, in one case, the moving object only uses one type of transportation tool of the entire trip, and in one case the sort of transportation tools is multiple from the starting point to the destination of the entire trip. Therefore, for a whole trajectory of moving object, the type of transportation mode during a period of data collection may be multiple or single, which may depend on the user’s travel purposes, income levels, the traffic condition etc. In this study, a trajectory generated by moving objects using one type or multi-type of transportation mode is classified as single-mode-based trajectory (SMT) or mixed-mode-based trajectory (MMT), respectively.

The classification of SMT or MMT based on underlying transportation mode is conducted around two tasks: transportation mode simplification and identification. Transportation mode simplification indicates to transform MMT to SMT by trajectory segmentation. Therefore, in many transportation mode detection studies, transportation mode simplification is the premise of mode detection especially for MMT. Then, for transportation mode identification of GPS data, the implementation mechanisms are changed because of specific algorithms, research area, and input-data [21,22,23,24,25]. In this study, the present status of mode classification methods for two kinds of trajectories (i.e., SMT and MMT) is reviewed in the following sections.

## 3. GPS Data Classification Based on the Transportation Mode

### 3.1. Overview

**Figure 3:**
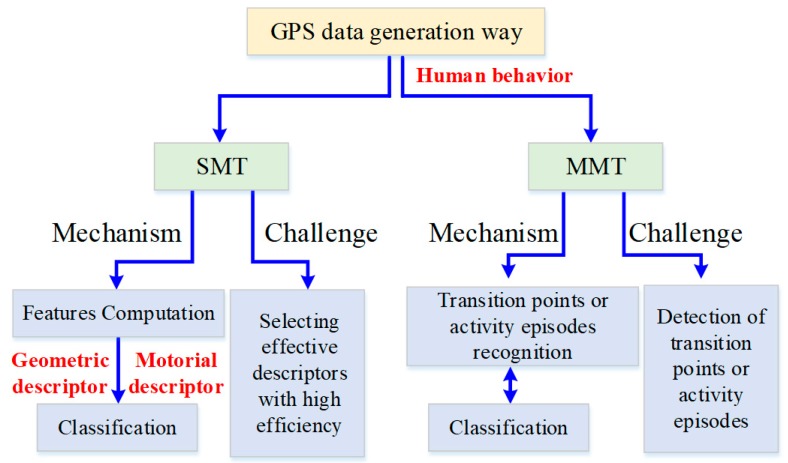
Overview of GPS data classification based on the transportation mode.

In this paper, GPS trajectories are classified into SMT and MMT based on the difference of GPS data generation way. The existing literatures are reviewed from these two sources of GPS trajectories, as shown in Figure 3:

Group 1: SMT is collected by one type of transport mode during moving, hence no segmentation is required. The mechanism of SMT classification based on transport mode has two main steps: features computation (also called movement parameters computation) and classification. The main challenge to a successful classification of all GPS data is how to select effective descriptors from a mass of features and improve the efficiency of classification, as shown in Figure 3.

Group 2: MMT is collected by multiple modes of transport, so it is necessary to recognize the transition points or activity episodes between modes (see Figure 3). Therefore, MMT classification, apart from the face of the same challenge of SMT classification, more time is needed to be addressed transition points or activity episodes recognition with high accuracy.

This section presents a detailed review of each group.

### 3.2. SMT Classification Based on Transportation Mode

The key task of SMT classification based on transport mode is to detect the transportation mode hiding in these data. On the basis of the previous studies, methods of transportation mode detection for SMT mainly have two steps: movement parameters computation and transportation mode classification. The movement parameters [31], also known as descriptors of trajectories, can be derived from the GPS trajectory and reflect the dynamic behavior of the moving object. Researchers proposed that movement parameters (e.g., speed and tortuosity, etc.) were used to identify trajectory segments of homogenous characteristics corresponding to particular behavioral states [32]. In general, the common movement parameters of GPS trajectories mainly include velocity, acceleration, turning angle, etc. Besides, the SMT is generated by one type of transport mode, the geometric shape of trajectories collected by different transportation tools maybe exist differences. For example, the geometric shape of pedestrian’s trajectories may be more complicated than vehicles’ trajectories, because they need to avoid obstacles when they are walking, while vehicles just need to travel along the road. Thus, some researchers also used sinuosity, tortuosity, or fractal dimension to quantify the geometric features of trajectories, besides speed, acceleration, and turning angle, etc. In this article, the parameters used to descript the geometrical characteristic of trajectories are termed as geometric descriptors (such as sinuosity, tortuosity and fractal dimension). The descriptions of movement such as speed, acceleration, turning angle are labeled as motorial descriptors, as shown in Table 2.

**Figure 4:**
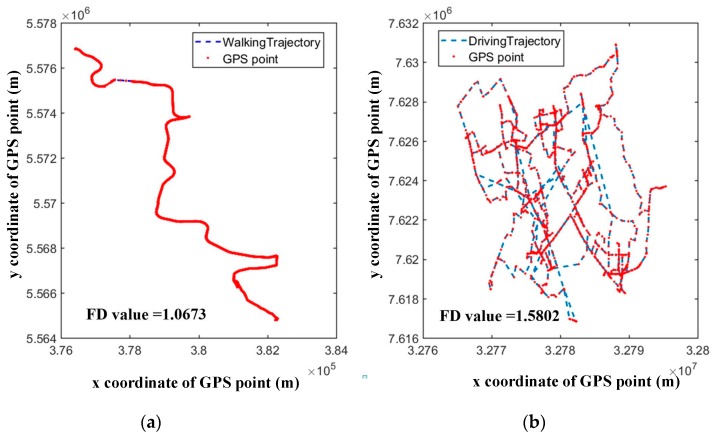
Fractal dimension values of trajectories. (**a**) Walking trajectory obtained from OSM website; (**b**) driving trajectories collected by taxis in Wuhan City.

Geometric descriptors (e.g., sinuosity, tortuosity, fractal dimension, etc.) of GPS trajectories are usually adopted to analyze animal moving pattern [39]. Inspired by geometric descriptors of animals’ moving trajectory, the author in Ref. [40] presents a novel trajectory classification model based on two types of complexity measures: geometric complexity and structural complexity. As stated in his article, the geometric shape of a trajectory can represent the characteristics of movement via its geometric complexity measure. So, he uses fractal dimension to measure the geometric complexity of trajectories. Although the value of the fractal dimension, sinuosity or straightness etc. could reflect the differences of transportation modes (e.g., walking and driving) to some degree, it is not robust for any situation. In the real world, the sinuosity of trajectories mainly depends on the moving behavior of moving objects and surroundings of movement. For example, sometimes the value of the fractal dimension of walking trajectory may be even lower than driving trajectory if the pedestrian keeps walking straight along the road, while the driver travels on a winding, maze-like streets, as shown in Figure 4. Besides, trajectories collected by car, bus, or motorbike share the same route network and show almost a similar sinuosity profile. Thus, in most studies, researchers didn’t take into the geometric descriptors account: authors in Ref. [41] proposed a vibration-based mode detection model using a Bayesian network with a focus on a fine distinction between a car and a motorbike. The main features used for classification are speed and acceleration of trajectories. In addition, researchers developed new point features: bearing rate and the change rate of the bearing rate to aid in classifying transportation mode by using machine learning techniques on the basis of primary study [42].

Training classifier is the next step of transportation mode detection of SMT. Based on the previous work, the Support Vector Machine (SVM) algorithm is the most common method for transportation mode classification of SMT [43]. During SMT classification process, to improve the operation efficiency, using correlation analysis to decrease the dimension of input features is also needed [33,34,35,36,37,38,39,40]. With the computer technology and statistical learning theory and its applications development, many new classification methods have also gained attraction: authors in Ref. [44] compared the performance of the traditional classification algorithms (K-Nearest Neighbor, Decision Tree, and Support Vector Machines) with the tree-based ensemble models (Random Forest, Gradient Boosting Decision Tree, and XGBoost) by using Geo-life data set. Among these machine learning algorithms, the experimental results shown that the XGBoost model produced the best performance. At the same time, a lot of movement parameters used for classification are ordered by importance based on the XGBoost model, which can be used to reduce the model complexity by removing the unimportant features. In addition to decision tree-based methods, neural network-based methods are also widely used in transportation mode estimation [45]. For example, some researchers integrated the hand-crafted features developed by article [46,47,48,49] with high-level features extracted through deep learning architectures. Authors in Ref. [46] proposed to detect travel modes of GPS data by using a Bayesian network of integrating with K2 algorithm and maximum likelihood methods. In Ref. [47], authors used a fully-connected Deep Neural Network (DNN) to automatically extract deep features and classify trajectories based on transportation mode. The core idea of their work was to convert GPS trajectories into 2-D image structures as the input for the DNN model. Similarly, researchers obtained the deep features by transforming point-level features with the aid of the sparse auto-encoder [48]. The deep features are then integrated with the hand-crafted features by using a simple Convolutional Neural Network (CNN). Researchers in Ref. [49] proposed to use CNN architectures to recognize transportation modes of GPS trajectories based on the studies of the limitation of the neural network-based method adopted past [47,48]. To emphasize an important point, the experimental data used in above methods are actually generated by MMT way but they first partition the entire trajectory into a series of segments with single transportation mode by using the label files. So they focus only on the improvement of classification accuracy of SMT by using the advanced machine learning techniques, the issues of transition point identification or trip segmentation have been overlooked.

### 3.3. MMT Classification Based on Transportation Mode

**Figure 5:**
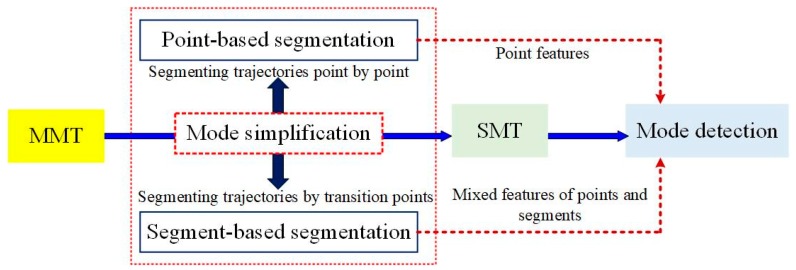
The mechanism of MMT classification based on transportation mode.

Compared to SMT, the classification of MMT based on the underlying transport mode is more complex because we need to not only segment trajectories, but also detect the transport mode implied in these segments. Thus, the task of MMT classification includes both transportation mode simplification and identification, as shown in Figure 5. 

To transform MMT to SMT, we first need to find the transitions between transportation modes. These behavioral transition points or change points represent the location where moving objects change their current transportation mode to another. In general, transition points can be detected by finding direction or speed changes [32,50,51,52,53,54,55,56,57], or special location points [58,59,60,61]. The detected transition points are then used to partition the whole trajectory into a series of sub-trajectories to ensure that each sub-trajectory is generated by one kind of transportation tool. Thus, for most of GPS data classification methods, the accuracy of transition point recognition directly affects the accuracy of transport mode classification results. Authors in Ref. [62] states that over-segmentation of the trajectories is better than under-segmentation since fast transitions between modes may pass undetected (e.g., exiting a tram/bus, and immediate departure with some other modes). In Ref. [63], researchers summarized that two different branches of science have focused on trajectories segmentation, which includes point-based and segment-based transportation segmentation. The point-based transportation segmentation is used to enrich applications and services with information derived from knowing a user’s transportation mode. Segment-based transportation segmentation is regarded as a way to automatically record the activity-travel diary of people. For MMT classification based on transportation mode, the differences of MMT segmentation also lead to different methods of transportation mode detection. Hence, from a data processing of view, the mechanism of MMT classification can also be reviewed from the aspects of point-based classification and segment-based classification, as shown in Figure 5. Detailed discussions for each kind of method are as follows.

#### 3.3.1. Point-Based Classification for MMT

From the view of the application, point-based transportation segmentation is usually used for real-time or near-real-time location-based services such as MoveSmarter [64], CO2GO (MIT Senseable City Lab), and MLANFIS [57]. Approaches to GPS data classification based on point-based segmentation mainly depend on a set of sophisticated features which are extracted from multi-sensors (e.g., GPS receiver, accelerometer, and gyroscope) or GIS database (e.g., road network, POI information). In Ref. [65], authors applied a hierarchical Markov model to infer a user’s daily movement with the information of bus stops, parking lots, and movement features extracted from GPS device. Authors in Ref. [66] proposed a method for real-time transportation mode detection by using the characteristics of the trail of GPS data of mobile phones. In their article, movement parameters including speed, acceleration, and a number of satellites in view are major input variables that are used to classify between different transportation modes. The neural network classifier is adopted to identify the mode based on the characteristics of GPS data streams. In Ref. [67], researchers presented an approach to inferring a user’s mode of transportation based on the GPS data generated by her mobile device and knowledge of the underlying transportation network. The transportation network information considered contains real time location information of bus, spatial rail, and spatial bus stop. As an improvement, authors in Ref. [68] also propose to use neural network-based artificial intelligence to identify the mode of transportation by detecting the patterns of the distinct physical profile of each mode that consists of speed, acceleration, number of satellites in view, and electromagnetic levels. Researchers developed a multi-layered neuro-fuzzy based model (MLANFIS) to detect transportation mode of GPS trajectories in near-real time by using motorial descriptors (e.g., average speed, maximum speed) and road network (e.g., bus network, tram network, and train network) [57]. In Ref. [69] the authors integrated the spatial (e.g., bus stop, road network) and temporal information (e.g., speed) with socio-demographic characteristics of travelers (e.g., personal preferences) to detect transportation mode by using dynamic Bayesian Networks. In addition, a support vectors machines-based model was developed to detect five transport modes via a combination of spatial context (e.g., transport network information) and motorial features (e.g., speed) of human movements [70]. All these studies discussed how to classify the transportation mode of each GPS point, not only by using GPS data but also other sensor data and GIS related information.

From the perspective of GPS data classification based on transportation mode, most of studies selected to extract features directly from GPS devices in aiding mode identification [44,61,65,67,68,69,70]. However, from the view of real-time applications based on transport mode detection, the limitations of GPS information associated with the use of GPS sensors still exist. For instance, GPS information is not available in shielded areas (e.g., tunnels) and the GPS signals may be lost especially in high dense locations, which results in inaccurate position information. Therefore, for real time transportation mode detection, many methods are proposed to make use of the information extracted from other built-in sensors of smart devices (e.g., accelerator) rather than GPS sensor [71,72,73,74,75,76]. Moreover, the accuracies of transportation mode detection of moving object were compared by using features extracted from GPS device or other sensors except for GPS device, and the experimental results show that the accuracy of the former (by using GPS data) is higher than the latter (by abandoning GPS data) [77]. Then, the authors published another paper [77] which demonstrated how to detect transportation mode of moving object in real-time by using data obtained from smartphone sensors including accelerometer, gyroscope, and rotation vector based on machine learning techniques [43], as well as the methods reviewed in Ref. [25].

In conclusion, point-based transportation segmentation is the mainstream approach for real-time applications. For such real-time applications, GPS data collected by GPS sensors have two major roles: First, GPS data only provides location information and will not be used to detect the transportation mode; Second, GPS data not only gives location information but also provides movement features (e.g., speed) to aid in real-time transportation mode detection. Although point-based transportation segmentation by using multi-sensors or GIS information database performs well for real-time applications, GPS trajectories collected from the back-end database of these applications still face the challenges of transportation mode classification if they have no mode flag.

#### 3.3.2. Segment-Based Classification for MMT

**Figure 6:**
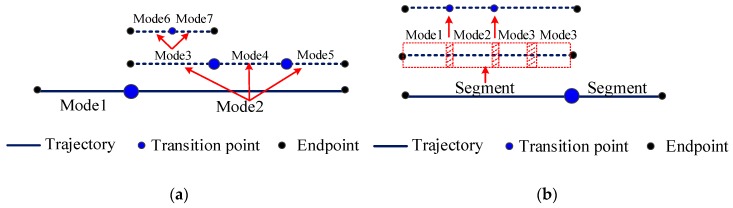
Transition point recognition of MMT. (**a**) Direct partitioning mode; (**b**) indirect partitioning mode.

In contrast to point-based transportation mode detection methods, approaches on segment-based transport mode detection from GPS data are generally not used for real-time location-based services. Because one of the characteristic features of segment-based transportation segmentation is that the mode detection results is time-delay. Thus segment-based transportation segmentation of GPS data is usually used for the research that is related to transportation-science (e.g., travel diary analysis) [78] or human-geography (e.g., human moving pattern mining [79]). For these applications, segment-based classification for MMT is usually taken as the data preprocessing to provide GPS data with specific transportation mode labels. In this study, we distinguish the methodology of segment-based classification of MMT based on the method of transition point recognition. On the basis of the previous study, we classify the methods of transition point recognition into two categories: *Direct partitioning mode* and *Indirect partitioning mode*, as shown in Figure 6. The corresponding methods of MMT classification are given in the following sections.

The *Direct partitioning mode* indicates that we need to find all the potential transition points first, and then using these potential transition points to partition the whole trajectory into a series of segments. To make sure each segment of MMT is collected by one type of transport tools, as shown in Figure 6a. Then, the transport mode of each segment of MMT is detected by the appropriate method and used for knowledge mining such as road network information, urban passenger transportation trans-shipment, moving behavior of urban residents, etc. For example, authors in Ref. [57] partitioned multi-modal trajectories by analyzing the proximity to potential transition points such as bus stops. In Ref. [39], authors proposed a trajectory decomposition algorithm, which includes two steps: global feature computation and local feature extraction. Especially, they computed the movement features (e.g., velocity, acceleration, turning angle, and straightness) for the entire trajectory. Then, they decomposed the profiles generated for different movement parameters using variations in sinuosity and deviation from the median line. This trajectory decomposition algorithm was applied to classify trajectories of moving objects based on transportation mode. The authors in Ref. [54] first used a change point-based segmentation method to partition each GPS trajectory into separate segments; then to classify the transport mode of each segment by using a set of sophisticated features which are extracted from GPS data based on the rules of human moving behavior. In Ref. [80], researchers segmented the trajectory by using the Spatio-Temporal Kernel Window (STKW) statistic. Specially, they ordered all trajectory points by time and then calculated the STKW values. The transition point between two modes was detected by analyzing the changes of STKW values of the trajectory.

Different from the above methods of *Direct partitioning mode*, a hierarchy model is introduced to classify the transportation mode of GPS data, which can improve the accuracy of classification result [62]. Based on the transport mode features, there are three layers of transportation modes. The first layer mainly includes three kinds of transport modes, that is, land transport, water transport and air transport. In the second layer, the land-transport is divided into the walk, bicycle, train, underground transport mode and motor vehicles. And water and air transport mode are respectively classified into boat and aircraft. In the third layer, the motor vehicles are furthermore classified into car, tram, and bus; and water transport mode: boat, is divided into ferry and sailing boat. Based on the method framework proposed in Ref. [62], the recognition of transition points is performed based on the layering of transport modes, and each layer corresponds to some specific rules. For example, for such transition point recognition, land-use information associated with the location of the trajectory is used in the first layer; the speed (e.g., nearly maximum speed, mean speed and mean moving speed), stop or signal shortage are used in the second layer; and the GIS information (e.g., bus stop) is used in the third layer. The fuzzy logic rules are used to classify the transportation mode of GPS data segment after transition point identification. Similarly, in Refs. [81,82], researchers identified transition point to classify transportation mode of GPS data through three steps. First, they used the concept of stops as location anchors to classify each record from valid GPS trajectories into stop segments and trip segments. Then, trip segments are divided into walk episodes and non-walk episodes based on the speed, heading, duration, and acceleration thresholds, etc. Finally, non-walk episodes are further classified into travel by car, bus or other modes based on probability model or machine learning method. Meanwhile, an enhanced transition point identification method [83] was proposed on the basis of the work in Ref. [79] to identify the transportation mode of segments by using Random Forest-based detection model. Unlike the study conducted by Ref. [84], authors in Ref. [83] first merge segments whose distance meets the threshold merely into its following segment instead of preceding and following segments to identify more transition points. Then, adopting iterative method in the segment collection to improve the accuracy of detecting transition point. Compared with the above studies, the study of transition point detection conducted by the authors of Ref. [85] was simpler. Since they just use time and distance thresholds and labeled files to segment the entire trajectories. Focus of their study is sought to select the most importance features from three sets of potential features extracted from trajectories during transportation mode detection.

In summary, the studies that rely on *Direct partitioning mode* show a common and effective way for MMT classification. However, the accuracy of transportation mode detection of MMT is too dependent on the transition point identification accuracy. In general, we can’t guarantee that all the transition points can be found because of many practical factors such as the precision of GPS data, the complexity of human behavior, the accuracy of GIS information, etc. Even if the uncertainty of transition point recognition can be reduced by using over-segmentation strategy, but too many fragments also cause the difficulty of subsequent transport mode detection of MMT classification.

The *Indirect portioning mode* for MMT classification is proposed to avoid the reliance on segmentation accuracy. The principle of *Indirect partitioning mode*, as opposed to *Direct partitioning mode*, that researchers need to detect transport mode first by using a moving window or thresholds (e.g., time, distance, speed) and then recognize transition point based on the detection results, as shown in Figure 6b. Case in point: in Ref. [86], researchers partitioned the trajectory into a series of segments by using the long gaps which are caused by the loss of GPS coverage due to indoor activity first. Then, a moving window-based SVM classification algorithm is used to detect the transport mode of each segment. A segmentation process is applied to the classified data after the initial SVM inference is performed. Besides, authors in Ref. [84] proposed to detect the transition point of transportation mode from trajectories based on three steps: candidate transition point identification, transportation modes prediction, and predicted modes modification. For candidate transition point identification, they first extracted the candidate transition points based on the velocity change and then merged all segments divided by candidate transition points with its nearby segments according to the specified distance threshold. Then, they used decision tree-based method to predict the transportation mode of each segment. Finally, the predicted modes sequence was modified based on logical assumption [51] as well as transition points. In contrast to *Direct partitioning mode*, *Indirect partitioning mode* can indeed reduce the reliance on partitioning accuracy. However, how to determine an optimal size of moving window, and how to minimize the impact of overlapping causing by moving window strategy to transportation detection are still challenges. Thus, the final decision for which method is more suitable should be made by considering many kinds of factors including the quality of GPS data, the richness and quality of additional information (e.g., data from other sensors or GIS information) and the aim of GPS data classification.

#### 3.3.3. Evaluation Indicators for GPS Data Classification Based on Transportation Mode

The evaluation of GPS data classification results is the core of the whole data classification system, which is also regarded as an important part for examining the effect and quality of the proposed classification method. In Ref. [21], authors compared the relative performance of different classification algorithms for the detection of transportation modes by using the same GPS data set. In this paper, the evaluation indicators are investigated according to the classification methods of SMT and MMT. Specially, for SMT classification, the indicators such as the precision, recall, and F1 score are used to evaluate the results ([40,41,47,48,49,87]). In contrast to MMT, each SMT is collected by one kind of transport mode, so researchers don’t need to consider how to evaluate the accuracy of transition point identification.

For MMT classification, no matter for point-based segmentation or segment-based segmentation, most transportation mode detection methods still use precision to evaluate the effectiveness of classification results. Table A1 and Table A2 shown in the appendix are compiled to provide more detailed and visual representation of the results evaluation, each table shows the comparison results of several typical examples from the aspects of data source, additional information (e.g., sensor data of other sensors, GIS information including bus stop, road network, etc.), movement features, classification algorithm and the precision of classification results. Based on the comparisons of Table A1 and Table A2, although the precisions of these researches show the performance of classification algorithms to some degree, we still can’t gain more information from them such as the accuracy of transition point identification. For example, in Table A1, we only know that the accuracy of classification results by using the method [65] is the highest in the field of point-based classification by using an unsupervised way. But that does not mean that their method is better than others with only one evaluation indicator. Beyond that, as Ref. [24] stated, the current error measures for point-based transportation mode detection methods cannot explain how well the methods describe the continuous transportation mode of the user during the studied time frame. Because they are insensitive for segments from the sequences of consecutive points of the same transportation mode. Moreover, the existing error measures for segment-based transportation segmentation do not respect the continuity of the transportation mode, and the matched segments cannot coincide with all ground truth segments. Therefore, to improve the drawbacks of the current error measures for MMT classification results, they proposed a novel error measure system for MMT based on the time algebra alignment. That is, except for precision, the evaluation indicators of their error measure system also include shift-in penalty, shift-out penalty and over-segmentation. By using those sophisticated indicators, researchers can better assess and investigate the performance of GPS data classification, and to find an optimal method for segmentation or classification based on the values of these evaluation indicators.

## 4. Discussion

Although the question of GPS data classification based on transportation mode has been discussed heatedly and most of the methods had high detection accuracy, there are still too many issues that needed to be addressed in the future work.

For SMT, one main problem is the homogeneity of the raw data set. That means each trajectory of the raw data set is gathered by only one kind of transportation tool. In the real world, however, most people will not automatically classify their moving trajectories according to their moving mode except for GPS data collected by public transportation system (e.g., taxi, bus, shared bike) based on the passive way. The similarities in some respects between transport modes make it hard for classification, such as riding with low speed and brisk walking. Besides that, from the view of the method, using shape complexity (e.g., Fractal dimension, tortuosity, sinuosity) to describe trajectories collected by different transportation modes (e.g., walking, driving) lacks robustness. Therefore, it is worth considering whether we should use geometric descriptor during SMT classification.

**Figure 7:**
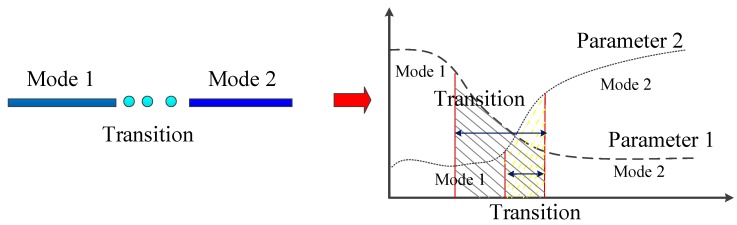
The transition between modes. The left panel shows a simplified graph of the transition from mode 1 to mode 2; the right panel shows the change of the value of corresponding parameters during transportation mode transition.

For MMT, there are two ways for classification based on the transportation mode, one is point-based mode and another is segment-based mode. The main issues of these two kinds of methods relate to the detection of changes/transition point. Based on the previous study, researchers use the additional information (e.g., parking lots, bus stop) to improve the accuracy of GPS data classification. Although the more information is used the higher precision will be got, the increase of the information on classification also proposed a higher demand for these processing tools with fast data analyzing especially for real-time services. Therefore, there is a trade-off between the information richness and the computation cost for point-based GPS data classification. Approaches to segment-based data classification generally include two patterns: *Direct partition mode* and *Indirect partition mode* based on the transition recognition methods. Compared with the methods of *Direct partition mode*, methods based on *Indirect partition mode* may be not limited by the accuracy of transition points identification. But the truth is that the change between modes is a process, that all movement parameters come a gradual change first and then stop the change after the previous mode has changed to another, as shown in Figure 7. The gradual change of mode transition increases the error of transportation mode classification especially for two similar adjacent transportation modes and makes it difficult to recognize the transition point based on the primary classifying result. There are two important limitations of existing transition point detection approaches: causing model misspecification and missing the continuous variations in the movement modes [88]. First, most of methods rely on a single, given movement parameter to identify behavioral change points. Thus, it is very difficult to identify cases where behavioral modes are affecting other parameters and causes model misspecification problem. Second, they focus on detecting abrupt changes and therefore missing the continuous variations in the movement modes. The research question about how to define the threshold of transition process during transportation mode detection still needs to be discussed.

The final issue of GPS data classification based on transportation mode is whether we should consider the context information during classification. Based on the previous studies, the context information of movement can be classified as human factors (e.g., age, occupation) and physical environment (e.g., location, background, weather) [89,90]. In general, people need to choose transportation mode according to the specific geographical environment [86]. For instance, we must walk in a mountainous area where there is no road for vehicles or bicycles. Beyond that, most people tend to walk, run or ride in a greener environment. So moving patterns like walking, running or bicycling may tend to happen in a park or near the mountain. At present, although some context information such as locations (e.g., bus stop, parking lots), backgrounds (e.g., land-use) have been used to recognize transportation mode of GPS data, a study which specializes in transportation detection from GPS data based on context-aware is still rare.

## 5. Conclusions

This paper presents an analysis of the research that surrounds GPS data classification based on transportation mode with an emphasis on how two modes of generation of GPS data, that is, SMT and MMT, approach this task. The paper is structured in three main parts that: Preliminaries, GPS data classification based on transportation mode, and Discussion. For the first part, the GPS data generation way corresponding with the type of GPS data travel mode is provided. The second part, GPS data classification, is the main body of this article. In this part, all existing studies for GPS data classification are reviewed based on the categories of GPS data generation way (SMT and MMT). Based on the study of GPS data classification, the biggest difference between SMT and MMT classification is whether it is necessary to find transition points between modes. Obviously, MMT classification needs to face more complicated problems than SMT, because transition point detection is required for it. Based on the transition point detection strategies for MMT, therefore, classification approaches are further divided into point-based and segment-based, and different types of method have approached this problem with different strategies. In addition, the review of evaluation indicators for GPS data classification is also discussed in this part. The last main part of the paper tries to discuss the issues of the present approaches for each type of GPS data classification based on transportation mode and identifies the most difficult challenges while doing so.

## Figures and Tables

**Table 1:** The comparison of GPS data collected from different ways.

	Position Precision	Real-Time	Movement Information
Passive Way	The precision of GPS data varies in a specific range and can be improved using automated quality algorithms. Data quality is ensured through standardized collection method.	High	Variable depending on application requirement
Active Way	The precision of GPS data varies in an unknown range. Data quality can’t be guaranteed.	Variable depending on level of engagement	Variable depending on users’ behavior

**Table 2:** The motorial and geometric descriptors for GPS trajectories.

Motorial descriptors	1. Speed (average, standard deviation, median value, skewness, approximate entropy, frequency)
	2. Acceleration (average, standard deviation, median value, skewness, approximate entropy, frequency)
	3. Turning angle/azimuth/heading (average, standard deviation, median value, skewness, approximate entropy, frequency)
	4. Distance (average, standard deviation, median value, skewness, approximate entropy, frequency) [32]
	5. First passage-time [33]
Geometric descriptors	6. Straightness (multi-scale) [34]
	7. Straightness index (multi-scale) [35] 8. Sinuosity/tortuosity of multi-scale [36,37,38,39]
	9. Fractal dimension

## Appendix A

**Table A1:** MMT classification evaluation based on point-based mode.

Method	GPS Data Source	Additional Information	Movement Features	Classification Algorithm	Precision
Ref. [61]	Collected 60 days of GPS data from one person	POI information (bus stops and parking lots)	Location; Velocity; Direction	Hierarchical Markov model (unsupervised learning)	98%
Ref. [62]	GPS device built-in Smart phone	no	Speed; Acceleration; Number of satellites	Neural networks (supervised learning)	82%
Ref. [63]	GPS device built-in Smart phone	Real-time bus locations; spatial rail and spatial bus stop	GPS data precision; Speed; Heading; Acceleration	Bayesian Net; Decision Tree; Random Forest; Naïve Bayesian and Multilayer Perceptron	93.5% (Random Forest)
Ref. [64]	GPS device built-in Smart phone	Sensor data from accelerometer and magnetometer	Speed; Acceleration; Number of satellites; Electromagnetic levels	Neural network-based artificial intelligence (supervised learning)	85%
Ref. [54]	GPS data collected by Android-based smartphone	Bus, train, and tram network	Average speed, maximum speed	Multi-layered neuro-fuzzy based model (MLANFIS)	83%
Ref. [65]	GPS devices built-in smart phone	Bus stops, rail stations, road network, socio-demographic characteristics of travelers	speed	Dynamic Bayesian Networks (Unsupervised classification)	72.5%
Ref. [66]	GPS devices built-in smartphone	Railway, motorway, charging stations, public transport stops	speed	Support Vectors machines-based model	94%

**Table A2:** MMT classification evaluation based on point-based mode.

Method	GPS Data Source	Additional Information	Movement Features	Classification Algorithm	Precision
Ref. [55]	4 months of GPS data by one person; collected by 5 participants in 1 week	bus stops and parking lots	Location; Velocity; Direction	Bayesian network (supervised learning)	80%
Ref. [36]	Public data of OSM	no	Velocity; acceleration; turning angle; straightness;	SVM (supervised learning)	94%
Ref. [51]	Collected by 65 users by using GPS-enabled device	no	Distance; Speed; Acceleration; Heading; Stop	Decision Tree-based inference model (supervised learning)	75%
Ref. [58]	Public data on OSM website	Bus station	Stop; Signal shortage; Speed; Distance;	Fuzzy logic concept (supervised learning)	91.6%
Ref. [81]	Bus traces were acquired from Inovative Tampere Site’s Journey APIs; other trajectories were acquired from the OSM and Geolife projects	no	Speed, Acceleration	Random forest	88.5%
Ref. [71]	Public data of Geo-life	Bus station	Velocity category, Acceleration category, Behavior category (e.g., bus stop rate)	DT and five kinds of DT-based combinatorial classification method	86.5%
Ref. [77]	GPS dataset from the Space-Time Activity Research project in Halifax, Canada	no	Median speed, median change in heading, total duration	Multinomial logit model	90%
Ref. [82]	Collected by 81 participants in two-weeks	no	Distance; Speed; Acceleration; Heading	SVM (supervised learning)	88%
Ref. [80]	Public data of Geo-life	no	Time-slice type, Acceleration change rate, Velocity, Acceleration, VCR, SR, HCR	Random Forest (supervised learning)	82.85%

## References

[1] Chon J., Cha H. (2011). Lifemap: A smartphone-based context provider for location-based services. IEEE Pervasive Comput..

[2] Chatzimilioudis G., Konstantinidis A., Laoudias C., Zeinalipour-Yazti D. (2012). Crowdsourcing with smartphones. IEEE Internet Comput..

[3] Kitchin R. (2014). The real-time city? Big data and smart urbanism. GeoJournal.

[4] Realini E., Caldera S., Pertusini L. (2017). Precise GNSS Positioning Using Smart Devices. Sensors.

[5] Odolinski R., Teunissen P.J.G. (2018). An assessment of smartphone and low-cost multi-GNSS single-frequency RTK positioning for low, medium and high ionospheric disturbance periods. J. Geod..

[6] Phithakkitnukoon S., Horanont T., Di Lorenzo G., Shibasaki R., Ratti C. (2010). Activity-aware map: Identifying human daily activity pattern using mobile phone data. Hum. Behav. Underst..

[7] Hu X., An S., Wang J. (2018). Taxi driver’s operation behavior and passengers’ demand analysis based on GPS data. J. Adv. Transp..

[8] Hassel D.V., Velden L.V.D., Bakker D.D., Batenburg R. (2017). Age-related differences in working hours among male and female GPS: An SMS-based time use study. Hum. Resour. Health.

[9] Nethery E., Mallach G., Rainham D., Goldberg M.S., Wheeler A.J. (2014). Using Global Positioning Systems (GPS) and temperature data to generate time-activity classifications for estimating personal exposure in air monitoring studies: An automated method. Environ. Health.

[10] Yang X., Tang L., Niu L., Zhang X., Li Q. (2018). Generating lane-based intersection maps from crowdsourcing big trace data. Transp. Res. Part C Emerg. Technol..

[11] Yang X., Tang L., Stewart K., Dong Z., Zhang X., Li Q. (2017). Automatic change detection in lane-level road networks using GPS trajectories. Int. J. Geogr. Inf. Sci..

[12] Tang L., Yang X., Dong Z., Li Q. (2016). CLRIC: Collecting lane-based road information via crowdsourcing. IEEE Trans. Intell. Transp. Syst..

[13] Tang L., Kan Z., Zhang X., Yang X., Huang F., Li Q. (2016). Travel time estimation at intersections based on low-frequency spatial-temporal GPS trajectory big data. Cartogr. Geogr. Inf. Sci..

[14] Pan G., Qi G., Wu Z., Zhang D., Li S. (2013). Land-use classification using taxi GPS trajectories. IEEE Trans. Intell. Transp. Syst..

[15] Tu W., Cao J., Yue Y., Shaw S.-L., Zhou M., Wang Z., Chang X., Xu Y., Li Q. (2017). Coupling mobile phone and social media data: A new approach to understanding urban functions and diurnal patterns. Int. J. Geogr. Inf. Sci..

[16] Yang J., Dong J., Hu L. (2017). A data-driven optimization-based approach for siting and sizing of electric taxi charging station. Transp. Res. Part C Emerg. Technol..

[17] Bao J., Zheng Y., Wilkie D., Mokbel M. (2015). Recommendations in location-based social networks: A survey. GeoInformatica.

[18] Cui G., Luo J., Wang X. (2018). Personalized travel route recommendation using collaborative filtering based on GPS trajectories. Int. J. Digit. Earth.

[19] Jean Damascène M., Timpf S. (2016). Trajectory data mining: A review of methods and applications. J. Spat. Inf. Sci..

[20] Pablo Samuel C., Zhang D., Chen C., Li S., Pan G. (2013). From taxi GPS traces to social and community dynamics: A survey. ACM Comput. Surv..

[21] Tao F., Harry J.P. (2016). Timmermans. Comparison of advanced imputation algorithms for detection of transportation mode and activity episode using GPS data. Transp. Plan. Technol..

[22] Shen L., Stopher P.R. (2014). Review of GPS travel survey and GPS data-processing methods. Transp. Rev..

[23] Gong L., Morikawa T., Yamamoto T., Sato H. (2014). Deriving personal trip data from GPS data: A literature review on the existing methodologies. Procedia Soc. Behav. Sci..

[24] Prelipcean A.C., Gidófalvi G., Susilo Y.O. (2017). Transportation mode detection—An in-depth review of applicability and reliability. Transp. Rev..

[25] Marija N., Bierlaire M. Review of transportation mode detection approaches based on smartphone data. Proceedings of the 17th Swiss Transport Research Conference.

[26] Yang X., Tang L., Zhang X., Li Q. (2018). A Data Cleaning Method for Big Trace Data Using Movement Consistency. Sensors.

[27] Lane N.D., Eisenman S.B., Musolesi M., Miluzzo E., Campbell A.T. (11–16). Urban sensing systems: Opportunistic or participatory? In Proceedings of the 9th ACM Workshop on Mobile Computing Systems and Applications, Napa Valley, CA, USA, 25–26 February 2008; pp.

[28] Harris D., Smith D., O’Neil C., Severinsen J. The role of real-time crowdsourced information and technology in supporting traveller information and network efficiency. Proceedings of the Automated Vehicles Symposium.

[29] Haklay M., Weber P. (2008). Openstreetmap: User-generated street maps. IEEE Pervasive Comput..

[30] Zheng Y., Chen Y., Li Q., Xie X., Ma W.Y. (2010). Understanding transportation modes based on GPS data for web applications. ACM Trans. Web.

[31] Buchin M., Driemel A., van Kreveld M., Sacristan V. (2011). Segmenting trajectories: A framework and algorithms using spatiotemporal criteria. J. Spat. Inf. Sci..

[32] Soleymani A., Pennekamp F., Dodge S., Weibel R. (2017). Characterizing change points and continuous transitions in movement behaviours using wavelet decomposition. Methods Ecol. Evol..

[33] Soleymani A., Cachat J., Robinson K., Dodge S., Kalueff A., Weibel R. (2014). Integrating cross-scale analysis in the spatial and temporal domains for classification of behavioral movement. J. Spat. Inf. Sci..

[34] Soleymani A., Van Loon E.E., Robert W. Capability of movement features extracted from GPS trajectories for the classification of fine-grained behaviors. Connecting a Digital Europe through Location and Place. Proceedings of the AGILE’2014 International Conference on Geographic Information Science.

[35] Bovet P., Benhamou S. (1991). Optimal sinuosity in central place foraging movements. Anim. Behav..

[36] Fisher N.I. (1993). Statistical Analysis of Circular Data.

[37] Benhamou S. (2004). How to reliably estimate the tortuosity of an animal’s path: Straightness, sinuosity, or fractal dimension?. J. Theor. Biol..

[38] Dodge S., Weibel R., Forootan E. (2009). Revealing the physics of movement: Comparing the similarity of movement characteristics of different types of moving objects. Comput. Environ. Urban Syst..

[39] Nams V.O. (2005). Using animal movement paths to measure response to spatial scale. Oecologia.

[40] Li X. (2014). Using complexity measures of movement for automatically detecting movement types of unknown GPS trajectories. Am. J. Geogr. Inf. Syst..

[41] Ohashi H., Akiyama T., Yamamoto M., Sato A. (2013). Modality Classification Method Based on the Model of Vibration Generation while Vehicles are Running. Inf. Process. Soc. Jpn..

[42] Etemad M., Júnior A.S., Matwin S. Predicting Transportation Modes of GPS Trajectories using Feature Engineering and Noise Removal. Proceedings of the Canadian Conference on Artificial Intelligence.

[43] Jahangiri A., Rakha H.A. (2015). Applying machine learning techniques to transportation mode recognition using mobile phone sensor data. IEEE Trans. Intell. Transp. Syst..

[44] Zhu X., Li J., Liu Z., Yang F. Learning Transportation Mode Choice for Context-Aware Services with Directed-Graph-Guided Fused Lasso from GPS Trajectory Data. Proceedings of the IEEE International Conference on Web Services.

[45] Gonzalez P.A., Weinstein J.S., Barbeau S.J., Labrador M.A., Winters P.L., Georggi N.L., Perez R. (2010). Automating mode detection for travel behaviour analysis by using global positioning system senabled mobile phones and neural networks. IET Intell. Transp. Syst..

[46] Xiao G., Juan Z., Zhang C. (2015). Travel mode detection based on GPS track data and Bayesian networks. Comput. Environ. Urban Syst..

[47] Endo Y., Toda H., Nishida K., Kawanobe A. (2016). Deep Feature Extraction from Trajectories for Transportation Mode Estimation. Advances in Knowledge Discovery and Data Mining.

[48] Wang H., Liu G.J., Duan J., Zhang L. (2017). Detecting Transportation Modes Using Deep Neural Network. IEICE Trans. Inf. Syst..

[49] Dabiri S., Heaslip K. (2018). Inferring transportation modes from GPS trajectories using a convolutional neural network. Transp. Res. Part C Emerg. Technol..

[50] Mountain D., Raper J. Modelling human spatio-temporal behaviour: A challenge for location-based services. Proceedings of the 6th International Conference on Geocomputation.

[51] Zheng Y., Liu L., Wang L., Xie X. Learning transportation mode from raw GPS data for geographic applications on the web. Proceedings of the 17th International Conference on World Wide Web.

[52] Gurarie E., Andrews R.D., Laidre K.L. (2010). A novel method for identifying behavioural changes in animal movement data. Ecol. Lett..

[53] Schuessler N., Axhausen K.W. (2009). Processing Raw Data from Global Positioning Systems Without Additional Information. Transp. Res. Rec. J. Transp. Res. Board.

[54] Zheng Y., Xie X., Ma W.Y. (2010). Geolife: A collaborative social networking service among user, location and trajectory. IEEE Data Eng. Bull..

[55] Xiao G., Juan Z., Gao J. Inferring trip ends from GPS data based on smartphones in Shanghai. Proceedings of the Transportation Research Board 94th Annual Meeting.

[56] Dodge S., Laube P., Weibel R. (2012). Movement similarity assessment using symbolic representation of trajectories. Int. J. Geogr. Inf. Syst..

[57] Thiebault A., Tremblay Y. (2013). Splitting animal trajectories into fine-scale behaviorally consistent movement units: Breaking points relate to external stimuli in a foraging seabird. Behav. Ecol. Sociobiol..

[58] Das R., Winter S. (2016). Detecting Urban Transport Modes Using a Hybrid Knowledge Driven Framework from GPS Trajectory. ISPRS Int. J. Geo-Inf..

[59] Liao L., Patterson D.J., Fox D., Kautz H. (2006). Building personal maps from GPS data. Ann. N. Y. Acad. Sci..

[60] Thierry B., Chaix B., Yan K. (2013). Detecting activity locations from raw GPS data: A novel kernel-based algorithm. Int. J. Health Geogr..

[61] Hwang S., Evans C., Hanke T.M. (2017). Detecting Stop Episodes from GPS Trajectories with Gaps. Seeing Cities Through Big Data.

[62] Biljecki F., Ledoux H., Van Oosterom P. (2013). Transportation mode-based segmentation and classification of movement trajectories. Int. J. Geogr. Inf. Sci..

[63] Prelipcean A.C., Gidofalvi G., Susilo Y.O. (2016). Measures of transport mode segmentation of trajectories. Int. J. Geogr. Inf. Sci..

[64] Geurs K.T., Thomas T., Bijlsma M., Douhou S. (2015). Automatic trip and mode detection with move smarter: First results from the dutch mobile mobility panel. Transp. Res. Procedia.

[65] Liao L., Patterson D.J., Fox D., Kautz H. (2007). Learning and inferring transportation routines. Artif. Intell..

[66] Byon Y.J., Abdulhai B., Shalaby A. (2009). Real-time transportation mode detection via tracking global positioning system mobile devices. J. Intell. Transp. Syst..

[67] Stenneth L., Wolfson O., Yu P.S., Xu B. Transportation mode detection using mobile phones and GIS information. Proceedings of the 19th ACM SIGSPATIAL International Conference on Advances in Geographic Information Systems.

[68] Byon Y.J., Liang S. (2014). Real-time transportation mode detection using smartphones and artificial neural networks: Performance comparisons between smartphones and conventional global positioning system sensors. J. Intell. Transp. Syst..

[69] Bantis T., Haworth J. (2017). Who you are is how you travel: A framework for transportation mode detection using individual and environmental characteristics. Transp. Res. Part C Emerg. Technol..

[70] Semanjski I., Gautama S., Ahas R., Witlox F. (2017). Spatial context mining approach for transport mode recognition from mobile sensed big data. Comput. Environ. Urban Syst..

[71] Nick T., Coersmeier E., Geldmacher J., Goetze J. Classifying means of transportation using mobile sensor data. Proceedings of the 2010 IEEE International Joint Conference on Neural Networks (IJCNN).

[72] Feng T., Timmermans H.J. (2013). Transportation mode recognition using GPS and accelerometer data. Transp. Res. Part C Emerg. Technol..

[73] Hemminki S., Nurmi P., Tarkoma S. Accelerometer-based transportation mode detection on smartphones. Proceedings of the 11th ACM Conference on Embedded Networked Sensor Systems.

[74] Shin D., Aliaga D., Tunçer B., Arisona S.M., Kim S., Zünd D., Schmitt G. (2015). Urban sensing: Using smartphones for transportation mode classification. Comput. Environ. Urban Syst..

[75] Shafique M.A., Hato E. (2015). Use of acceleration data for transportation mode prediction. Transportation.

[76] Lan G., Xu W., Khalifa S., Hassan M., Hu W. Transportation mode detection using kinetic energy harvesting wearables. Proceedings of the IEEE International Conference on Pervasive Computing and Communication Workshops.

[77] Jahangiri A., Rakha H. Developing a support vector machine (SVM) classifier for transportation mode identification by using mobile phone sensor data. Proceedings of the Transportation Research Board 93rd Annual Meeting.

[78] Zhao F., Ghorpade A., Pereira F.C., Zegras C., Ben-Akiva M. (2015). Stop detection in smartphone-based travel surveys. Transp. Res. Procedia.

[79] Gautama S., Atzmueller M., Kostakos V., Gillis D., Hosio S. (2017). Observing Human Activity Through Sensing. Participatory Sensing, Opinions and Collective Awareness.

[80] Siłanowicka K., Vandrol J., Oshan T. (2016). Analysis of human mobility patterns from GPS trajectories and contextual information. Int. J. Geogr. Inf. Sci..

[81] Dalumpines R., Scott D.M. (2017). Making mode detection transferable: Extracting activity and travel episodes from GPS data using the multinomial logit model and python. Transp. Plan. Technol..

[82] Zheng Y., Wang L., Liu L., Xie X. (2017). Learning Transportation Modes from Raw GPS Data. U.S. Patent.

[83] Liang J., Zhu Q., Zhu M., Li M., Li X., Wang J., You S., Zhang Y. An enhanced transportation mode detection method based on GPS data. Proceedings of the International Conference of Pioneering Computer Scientists, Engineers and Educators.

[84] Zhu Q., Zhu M., Li M., Fu M., Huang Z., Gan Q., Zhou Z. (2016). Identifying transportation modes from raw GPS data. Communications in Computer and Information Science, Proceedings of the International Conference of Pioneering Computer Scientists, Engineers and Educators, Harbin, China, 20–22 August 2016.

[85] Mäenpää H., Lobov A., Lastra J.L.M. (2017). Travel mode estimation for multi-modal journey planner. Transp. Res. Part C Emerg. Technol..

[86] Bolbol A., Cheng T., Tsapakis I., Haworth J. (2012). Inferring hybrid transportation modes from sparse GPS data using a moving window SVM classification. Comput. Environ. Urban Syst..

[87] Xiao Z., Wang Y., Fu K., Wu F. (2017). Identifying Different Transportation Modes from Trajectory Data Using Tree-Based Ensemble Classifiers. ISPRS Int. J. Geo-Inf..

[88] Gurarie E., Bracis C., Delgado M., Meckley T.D., Kojola I., Wagner C.M. (2016). What is the animal doing? Tools for exploring behavioural structure in animal movements. J. Anim. Ecol..

[89] Geng X., Smith-Miles K., Wang L., Li M., Wu Q. (2010). Context-aware fusion: A case study on fusion of gait and face for human identification in video. Pattern Recognit..

[90] Brum-Bastos V.S., Long J.A., Demšar U. Dynamic trajectory annotation for integrating environmental and movement data. Proceedings of the Visually-Supported Computational Movement Analysis Workshop-AGILE.

